# Spatial Positioning of Immune Hotspots Reflects the Interplay between B and T Cells in Lung Squamous Cell Carcinoma

**DOI:** 10.1158/0008-5472.CAN-22-2589

**Published:** 2023-02-28

**Authors:** Hanyun Zhang, Khalid AbdulJabbar, David A. Moore, Ayse Akarca, Katey S.S. Enfield, Mariam Jamal-Hanjani, Shan E. Ahmed Raza, Selvaraju Veeriah, Roberto Salgado, Nicholas McGranahan, John Le Quesne, Charles Swanton, Teresa Marafioti, Yinyin Yuan

**Affiliations:** 1 Centre for Evolution and Cancer, The Institute of Cancer Research, London, United Kingdom.; 2 Division of Molecular Pathology, The Institute of Cancer Research, London, United Kingdom.; 3 Cancer Research UK Lung Cancer Centre of Excellence, University College London Cancer Institute, London, United Kingdom.; 4 Department of Cellular Pathology, University College London Hospitals, London, United Kingdom.; 5 Cancer Evolution and Genome Instability Laboratory, The Francis Crick Institute, London, United Kingdom.; 6 Department of Oncology, University College London Hospitals, London, United Kingdom.; 7 Cancer Metastasis Lab, University College London Cancer Institute, London, United Kingdom.; 8 Department of Pathology, ZAS Hospitals, Antwerp, Belgium.; 9 Division of Research, Peter MacCallum Cancer Centre, University of Melbourne, Melbourne, Victoria, Australia.; 10 Cancer Genome Evolution Research Group, Cancer Research UK Lung Cancer Centre of Excellence, University College London Cancer Institute, London, United Kingdom.; 11 Cancer Research UK Beatson Institute, Glasgow, United Kingdom.; 12 School of Cancer Sciences, University of Glasgow, Glasgow, United Kingdom.; 13 NHS Greater Glasgow and Clyde Pathology Department, Queen Elizabeth University Hospital, London, United Kingdom.

## Abstract

Beyond tertiary lymphoid structures, a significant number of immune-rich areas without germinal center-like structures are observed in non–small cell lung cancer. Here, we integrated transcriptomic data and digital pathology images to study the prognostic implications, spatial locations, and constitution of immune rich areas (immune hotspots) in a cohort of 935 patients with lung cancer from The Cancer Genome Atlas. A high intratumoral immune hotspot score, which measures the proportion of immune hotspots interfacing with tumor islands, was correlated with poor overall survival in lung squamous cell carcinoma but not in lung adenocarcinoma. Lung squamous cell carcinomas with high intratumoral immune hotspot scores were characterized by consistent upregulation of B-cell signatures. Spatial statistical analyses conducted on serial multiplex IHC slides further revealed that only 4.87% of peritumoral immune hotspots and 0.26% of intratumoral immune hotspots were tertiary lymphoid structures. Significantly lower densities of CD20^+^CXCR5^+^ and CD79b^+^ B cells and less diverse immune cell interactions were found in intratumoral immune hotspots compared with peritumoral immune hotspots. Furthermore, there was a negative correlation between the percentages of CD8^+^ T cells and T regulatory cells in intratumoral but not in peritumoral immune hotspots, with tertiary lymphoid structures excluded. These findings suggest that the intratumoral immune hotspots reflect an immunosuppressive niche compared with peritumoral immune hotspots, independent of the distribution of tertiary lymphoid structures. A balance toward increased intratumoral immune hotspots is indicative of a compromised antitumor immune response and poor outcome in lung squamous cell carcinoma.

**Significance:**

Intratumoral immune hotspots beyond tertiary lymphoid structures reflect an immunosuppressive microenvironment, different from peritumoral immune hotspots, warranting further study in the context of immunotherapies.

## Introduction

Despite a plethora of research dedicated to the study of non–small cell lung cancer (NSCLC) intratumor heterogeneity, deficiencies in our knowledge of the fundamental changes that occur within the tumor microenvironment have impeded progress in this area. Diverse immune responses have been proposed for different NSCLC subtypes, lung adenocarcinoma (LUAD), and lung squamous cell carcinoma (LUSC) based on the analysis of genome and transcriptome (1). Our recent findings resolve the link between spatial variability of lymphocyte infiltration with tumor immune evasion in LUAD (2). However, it is unclear how diverse immune cell subsets cooperate in a compact niche to drive pro- or antitumor responses, or whether they can be effectively measured in histology samples as part of the clinical routine (3).

We have previously demonstrated the importance of studying tumor spatial architecture for defining the clinical relevance of the immune response in estrogen receptor–positive and -negative breast cancer (4, 5). Spatial aggregation, rather than the sheer abundance of lymphocytes, is associated with recurrence-free survival in both breast cancer subtypes (4, 5). While this highlights the importance of examining not just cell abundance but also spatial patterns that can inform disease prognosis, important questions remain: (i) whether our observation extends to other cancer types beyond breast cancer, such as the highly immunogenic NSCLC; (ii) how these spatial patterns translate into immune cell functions and functional phenotypes; and (iii) how they collectively drive a microenvironment that promotes or suppresses cancer progression. In this article, we used transcriptomic data and deep learning–based digital pathology image analysis to: (i) discover the clinical relevance of spatial organization of lymphocytes in NSCLC; (ii) identify the transcriptomics underpinning of immune spatial heterogeneity; and (iii) uncover the interplay of immune cell subsets underlying distinct spatial organization patterns.

## Materials and Methods

### Patients and cohorts

All NSCLC whole-tumor hematoxylin and eosin (H&E) sections from The Cancer Genome Atlas (TCGA) analyzed in this study were downloaded from the TCGA data portal (*n* = 743 LUAD and 712 LUSC). These sections correspond to the total of 473 patients in TCGA LUAD and 466 in TCGA LUSC. Overall survival data (i.e., times to death or follow-up and vital status) was available for 935 patients with NSCLC, whereas additional clinical data (stage, age, smoking pack-years) were only available for 723 patients with NSCLC. The multivariate Cox validation took into account equally distributed discovery (*n* = 237 LUAD and 231 LUSC) and validation (*n* = 236 LUAD and 231 LUSC) cohorts.

The 10 external validation samples with multiplex IHC staining were obtained from the Leicester Archival Thoracic Tumor Investigatory Cohort (LATTICe) study, which consists of 1700 University Hospitals of Leicester Trust patients who underwent surgical treatment with curative intent for primary lung tumors. A consultant thoracic malignancy pathologist visually examined 20 H&E slides corresponding to 20 patients with high immune clustering. All 20 cases were analyzed using the immune hotspot pipeline to rank the top 10 cases. These 10 cases were then dearchived, and three serial sections were prepared for T-cell, H&E, and B-cell staining. The validation cohort was ethically approved by an NHS research ethics committee (ref. 14/EM/1159).

### Automated H&E image analysis mapped heterogeneous cell populations

**Figure 1.**
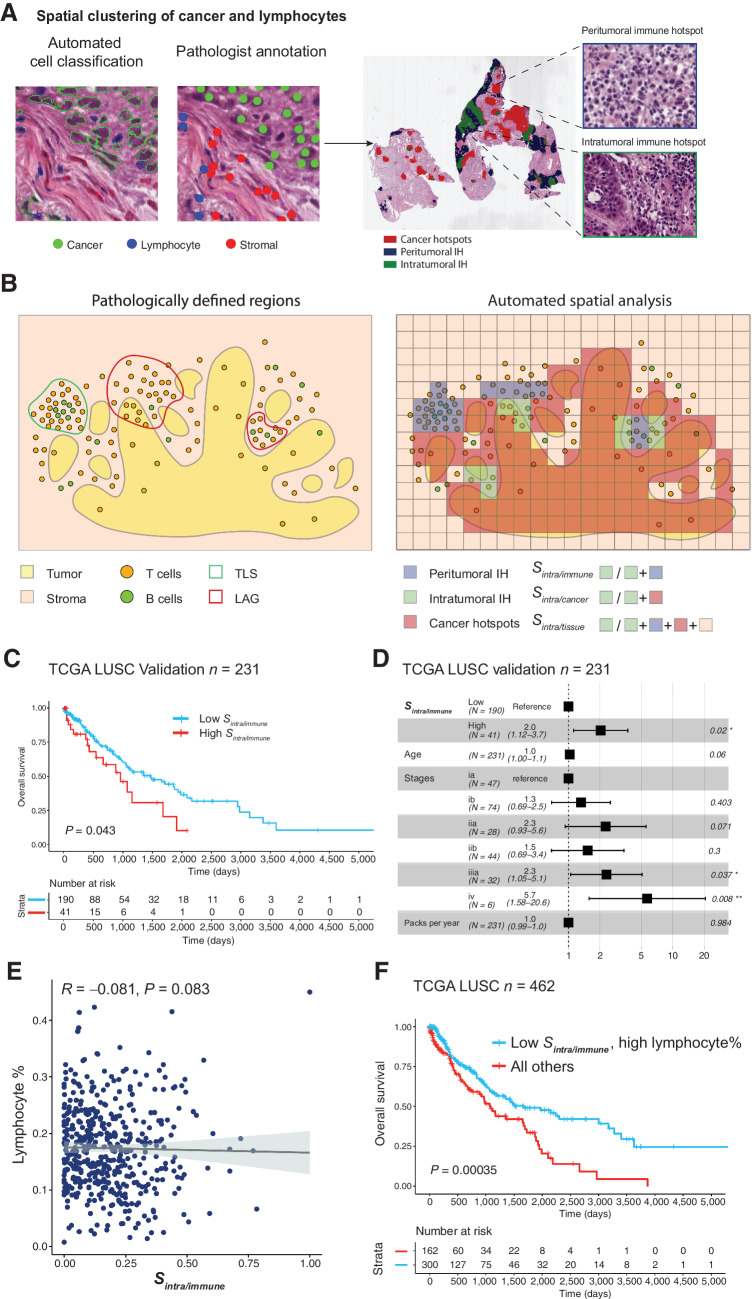
Immune hotspots in LUSC. **A,** Illustrative examples of automatic cell classification results and hotspot distribution with zoomed-in regions showing areas enriched with immune cells and cancer–immune coclustering. Cancer hotspots (red) and IHs were identified based on the local clustering of cancer and lymphocytes. Intratumoral IH (green) was determined as regions enriched with both immune and cancer cells, while peritumoral IH (blue) were regions enriched with only immune cells. **B,** Schematic representations of tissue compartments including cancer hotspots, peritumoral IH, intratumoral IH based on automated spatial analysis, and pathologically defined TLSs and lymphoid aggregates without clear separation of T- and B-cell zones (LAG). The three spatial scores *S*_*intra*/*immune*_, S_intra/cancer_, and S_intra/tissue_ were determined as the proportion of intratumoral IH in the immune-rich area (intratumoral IH and peritumoral IH), cancer-rich area (intratumoral IH and cancer hotspots), and the entire tissue, respectively. **C,** Kaplan–Meier curves to illustrate the difference in OS in LUSC patients stratified by S_intra/immune_, with the dichotomization optimized for the discovery cohort (*n* = 231) and assessed in the validation cohort (*n* = 231). **D,** Forest plots to show the prognostic value of *S*_*intra*/*immune*_ in multivariate models adjusted for stage, age, and smoking pack years in the validation cohort. **E,** Spearman correlation between lymphocyte percentage and *S*_*intra*/*immune*_ in the TCGA LUSC cohort (*n* = 462). **F,** Kaplan–Meier curve to illustrate the difference in OS in LUSC patients stratified by both S_intra/immune_ and lymphocyte percentage, with the stratification of the entire cohort shown (*n* = 462).

We collected four orthogonal data types to test the accuracy of automated H&E image analysis for single-cell classification in whole-section NSCLC images. CRImage (6) identified a total of 206,272,040 cancer cells, 35,733,365 stromal cells, and 49,953,400 lymphocytes in 1,455 H&E sections from frozen samples of 939 TCGA NSCLC patients. The balanced accuracy as an average of specification and sensitivity of the classifier was 82.13% for cancer cells, 89.16% for stromal cells and 87.81% for lymphocytes, comparing against 4,169 cancer, 1,533 stromal cells and 1,999 lymphocytes annotations by a thoracic malignancy pathologist in randomly sampled images (Fig. 1A). Automated cell percentage was more strongly correlated with molecular gene signatures than the independent scoring provided by TCGA pathologists (Supplementary Fig. S1A and S1C). This included a comparison between cancer cell percentages given by automated image analysis and pathologic scores of H&E, and tumor purity measures from the gene expression-based method ESTIMATE (7) and copy number–based ABSOLUTE (8). For stromal cell estimates, we compared H&E-derived scores with stromal scores from gene expression–based MCP-counter (9) and ESTIMATE (7). There was no pathologic score of lymphocytes available from the TCGA dataset, instead the lymphocyte percentage estimated by automated image analysis was evaluated against immune signatures inferred by TIMER (10). Automatic quantification of lymphocyte percentages significantly correlated with CD4, CD8 T-cell, and B-cell scores (*R* = 0.31, 0.17, and 0.24, respectively; *P* < 0.01; Supplementary Fig. S1B)

### Bioinformatic analysis

**Figure 2.**
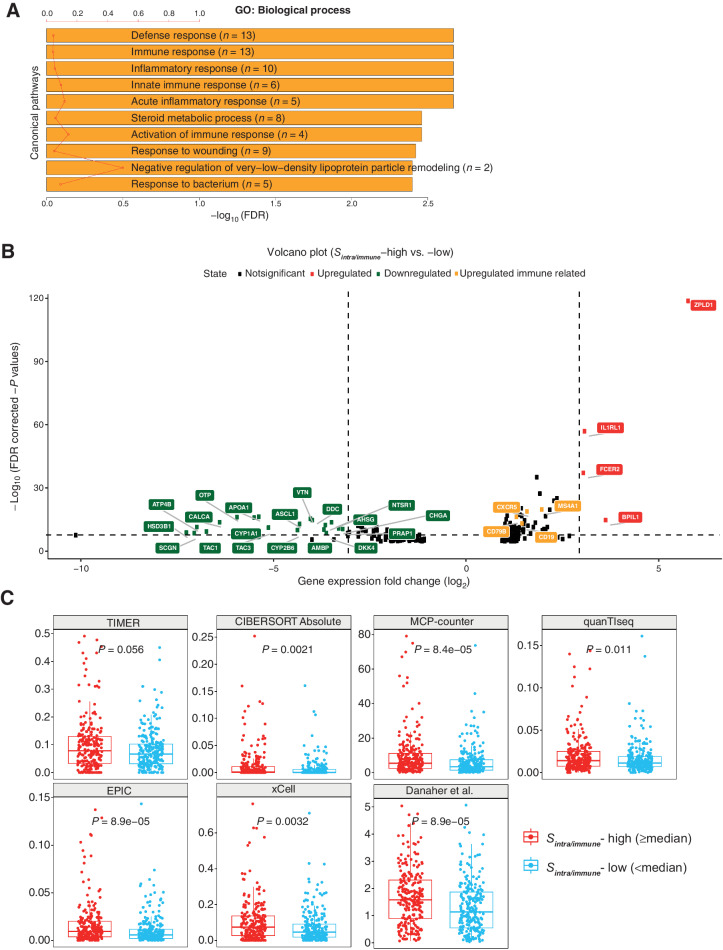
B-cell dominance of high-*S*_*intra*/*immune*_ group in LUSC. **A,** Gene Ontology (GO) biological processes found to be enriched in the DEGs according to patient groups stratified by *S*_*intra*/*immune*_. The red line denotes the ratio of the number of DEGs in a pathway to the total number of genes in that pathway. FDR, false discovery rate. **B,** Volcano plot showing DEGs in the high-*S*_*intra*/*immune*_ group compared with the low group. Genes with log-fold change (logFC) smaller than 1 were excluded. Genes involved in immune responses are highlighted in yellow. **C,** Consistently upregulated B-cell signatures in high-*S*_*intra*/*immune*_ group across seven bioinformatics methods using the TCGA LUSC cohort, *n* = 459. Statistical significance was evaluated by the Wilcoxon rank sum test and adjusted by the Benjamini–Hochberg method.

The gene enrichment analysis for the *S*_*intra*/*immune*_ in LUSC was performed using the “TCGAbiolinks” package in R. The RNA-sequencing data (all Level 3 raw read counts, available from the TCGA data portal) were used as input to the transcriptome differential gene expression analysis. The outputs of highly enriched genes clustered under the Gene Ontology biological processes for LUSC and LUAD are shown in Fig. 2A and Supplementary Fig. S2A, respectively. The highly enriched immune genes relevant to the immune response/activation of immune response processes in LUSC included *DEFB4A*, *LTF*, *FCN3*, *C7*, *MS4A1*, *PAX5*, *PLA2G1B*, *PLA2G2A*, *CHGA/CGA*, *FGB*, *BPIL1*, *DEFA5*, *CR2*, *PLUNC*, *C8B*, *DMBT1*, *KRT1*. The differential gene expression analysis was performed using the ‘DESeq2’ R package to rank differentially expressed genes with logFC larger than 1 in high versus low groups for the *S*_*intra*/*immune*_ in LUSC and LUAD, with the optimal cut-off point determined on the entire cohort (Supplementary Table S1; Fig. 2B; Supplementary Fig. S2B). The B-cell signatures were estimated by seven algorithms Danaher (11), TIMER (10), CIBERSORT Absolute mode (12), MCP-counter (9), quanTIseq (13), EPIC (14), and xCell (15). The patient-level estimates were calculated as the average across slide-level scores of the patient. Comparisons between *S*_*intra*/*immune*_ high and low patients stratified by the median revealed a consistent upregulation of B-cell infiltrates in the high group (Fig. 2C). The tumor mutation burden (TMB) was computed as somatic mutations per Mb using R library “maftools” (16) for 444 TCGA LUSC tumors with mutation annotation format files available. The clonal neoantigen load was derived from a previous work (1).

### Survival analysis of TCGA datasets

The investigated scores (*S*_*intra*/*immune*_, *S*_*intra*/*cancer*_, *S*_*intra*/*tissue*_) were first dichotomized using Kaplan–Meier curves (R “survminer” package) to find an optimal cut-point on the discovery cohort (*n* = 237 LUAD and 231 LUSC). The optimal cut-off point was computed with maximally selected log-rank statistics. Next, the dichotomized cut-off point found in discovery was used in a multivariate Cox proportional hazards model (R “survival” package) adjusted for stage, age, and smoking pack-years to further substantiate the survival prognosis. The Kaplan–Meier curve and the Cox model with the same cut-off point were then tested in the validation cohort (*n* = 236 LUAD and 231 LUSC), which revealed that the *S*_*intra*/*immune*_ is a LUSC-specific prognosticator. The survival analysis was repeatedly performed for four patient groups determined by their smoking history (current reformed smokers for less than 15 years, current reformed smokers for more than 15 years, current smokers and lifelong nonsmokers; *n* = 457 LUAD and 446 LUSC in total) with the cut-off point of *S*_*intra*/*immune*_ optimized for each category. Lymphocyte percentage and TMB were dichotomized using a cut-off point optimized for the discovery cohort. To evaluate the contingency of survival analysis on the split of cohorts, we randomly split the TCGA LUSC cohort into discovery and validation by a 1:1 ratio for 100 times. In each iteration, the numerical parameters (*S*_*intra*/*immune*_, age, pack years, and TMB) were dichotomized by the threshold optimized for the discovery cohort, followed by a log-rank test conducted in the validation cohort to determine the statistical difference in the OS of patients stratified by the dichotomized parameters. The stage was assessed as a categorical parameter in the validation cohort.

### Statistical analysis

Automated cell percentages and immune scores were correlated using Pearson method with the TCGA pathologic scores, molecular gene signatures, and gene expression–based scores (Supplementary Fig. S1B). Cell percentage in IHC slides was computed as the cell counts of each immune subset divided by the total number of six immune cell subsets identified on IHC sections. For statistical comparisons among groups, Wilcoxon test was used. All statistical tests were two-sided, a *P* value of less than 0.05 was considered statistically significant. All statistical analyses were conducted in *R* version 3.5.1.

### Identification of hotspots

Hotspot analysis was conducted on H&E slides using the method and parameters as previously proposed (5). Getis–Ord spatial analysis was carried out over 50×50 μm^2^ grids using the following equations:




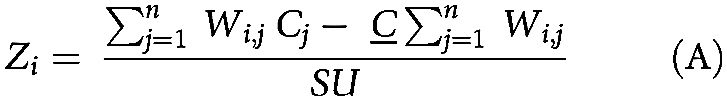







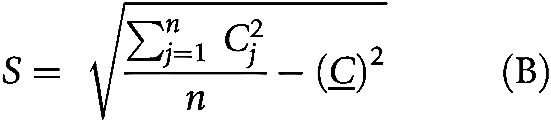







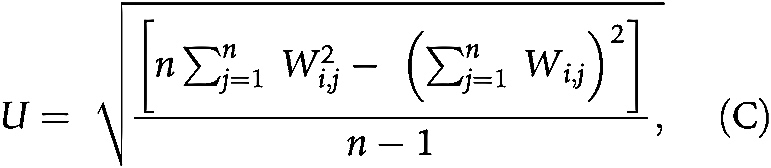




where *n* is the number of grids; *C_j_* is the count of cancer cells or lymphocytes in grid *j*; *W*_*i*,*j*_ is 1 if grid *i* and *j* are neighbors, otherwise 0. Grids sharing a vertex or edge with the analyzed grid *j* were defined as first-order neighbors. Second-order neighbors included grids sharing a vertex or edge with the first order neighbors and so on. Grids within the fourth order neighborhood were considered neighbors to grid *j* in the equation (5). P value was calculated on the basis of Z score thresholds (17). Regions with *P* < 0.05 regarding cancer cells and *P*  ≥ 0.05 regarding lymphocytes were categorized as cancer hotspots (CH). Regions with *P* < 0.05 regarding lymphocytes and *P* ≥ 0.05 regarding cancer cells were categorized as peritumoral immune hotspot (*IH_peri_*). And intratumoral immune hotspot (*IH_intra_*) was defined as regions with *P* < 0.05 for both cancer cells and lymphocytes. The definitions of *IH_peri_* and *IH_intra_* were described by the following equations.




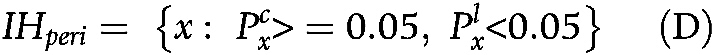







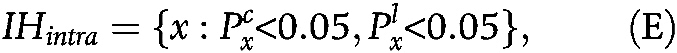




where $P_x^c$ and $P_x^l$ represent the *P* value quantifying the significance level of enrichment of cancer cells and lymphocytes in grid *x*, respectively.

To quantify the spatial relationship between immune and cancer aggregates for each tumor, we derived three scores based on different normalization terms for the *IH_intra_* (5).

1. To quantify the proportion of IHs interfacing with tumor, the intratumoral immune hotspot score *S*_*intra*/*immune*_ was computed as the proportion of *IH_intra_* in the immune cell rich area.
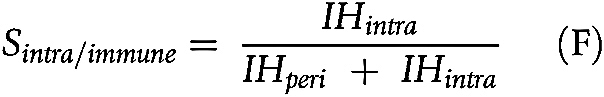
2. To measure the amount of intratumoral immune aggregates in relation to the cancer area, *S*_*intra*/*cancer*_ was computed as the ratio of *IH_intra_* to the sum of CH and *IH_intra_*.
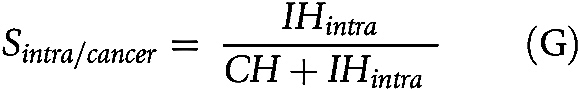
3. To quantify the colocalization of immune and cancer aggregates in the context of the entire tissue, *S*_*intra*/*tissue*_ was computed as the fraction of *IH_intra_* in the whole tissue including both hotspot area and non-hotspot area.
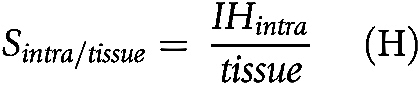

### Multiplex IHC

Diagnostic blocks were obtained for 10 patients with LUSC in the validation cohort. Tissue blocks were fixed with formalin, embedded in paraffin, then serially sectioned into sections of 4-μm in thickness. Serial sections from the validation cohort were subjected to H&E staining and multiplex IHC using the T-cell panel: anti-CD8 (type: mouse monoclonal, clone: 4B11, source: Leica Microsystems Ltd., used at 1:100 dilution); anti-CD4 (type: mouse monoclonal, clone name: 4B12, source: Leica Microsystems Ltd., used at 1:50 dilution); anti-FOXP3 [type: mouse monoclonal, clone: 236A/E3, source: kindly gifted by Dr. G. Roncador (CNIO, Madrid, Spain), used at 1:2 dilution] and the B-cell panel: anti-CXCR5 (type: rabbit polyclonal, catalog no. ABN200, source: Merck Life Science UK Limited, used at 1:1,000 dilution); anti-CD79b (type: mouse monoclonal, clone: B29/123, source: LRF Immunodiagnostics Unit, John Radcliffe Hospital, used at 1:25 dilution); anti-CD20 (type: mouse monoclonal, clone: L26, source: Agilent Technologies LDA UK Ltd., used at 1:100 dilution); anti-P40 (type: mouse monoclonal, clone: BC28, source: Biocare Medical, used at 1:100 dilution). All slides were scanned using the NanoZoomer S210 digital slide scanner (C13239–01) and the NanoZoomer digital pathology system v.3.1.7 (Hamamatsu) at ×40 (228 nm/pixel resolution).

### Deep learning pipeline to map cell populations on H&E and IHC images of the external validation samples

We performed cell detection and classification on H&E slides and the IHC T-cell panel of the validation cohort using the SCCNN pipeline (2, 18) pretrained on H&E and IHC images, respectively. Because of variations in the staining protocol, we trained an additional SCCNN-IHC classifier to discriminate CD4^+^FOXP3^−^ and CD4^+^FOXP3^+^ cells from this cohort. The SCCNN pipeline consists of two models to perform cell detection and classification sequentially. The cell detection was performed on 31×31 pixels image patches to predict pixels with high probabilities of being the cell center. The output is a table of predicted cell coordinates without class label. The table is then used to retrieve single cell images of 51×51 pixels centering at the predicted cell location. The second step involves classifying nine neighboring locations near the center of the cell. The most frequent class predicted among the nine neighborhoods will be determined as the class of the cell (2, 18).

Prior to the model implementation, whole slide images of H&E and IHC samples were rescaled to 0.45-μm/pixel and divided into tiles of 2000×2000 pixels to match the resolution of training images of the SCCNN. A color normalization step was performed for H&E tiles using an example H&E tile from the training set as reference. The H&E tiles were first converted from RGB to Lab color space. Then a Reinhard stain normalization from the Image processing toolbox in Matlab R2019b (RRID:SCR_001622) was used to transform all the H&E tiles based on the reference image. We did not normalize the color for the IHC slides. Instead, the classifier was trained on single cell patches augmented by contrast and brightness adjustment, which encouraged the model to capture stain variability in the dataset.

For the B-cell panel, we trained a distance regularized dense inception network (DRDIN; ref. 19) for cell detection using masks of annotated cell locations, and an SCCNN-IHC classifier (18) to differentiate between three dominant B-cell subsets (CD20^+^CXCR5^−^, CD20^+^CXCR5^+^, and CD79b^+^), and the P40^+^ neoplastic cells. Similar to SCCNN detector, the DRDIN model was trained to predict pixels of the cell center (19). However, the input image size was of 224×224 pixels, allowing the integration of features in a larger spatial context and therefore a better capability to capture densely located cells compared with SCCNN. Models for the B-cell panel were developed on a total of 13,545 single-cell annotations collected from 5 of 10 IHC slides and randomly split into training (64%), validation (16%), and testing (20%). Cell annotations of the T-cell and B-cell panels were both generated by A. Akarca and H. Zhang, and reviewed by T. Marafioti. To ensure the quality of cell annotations, we excluded labels disagreed by the two annotators from the dataset.

On average, we identified 69,146 ± 22908 CD8^+^, 109,764 ± 39,028 CD4^+^FOXP3^−^ and 34,861 ± 28,737 CD4^+^FOXP3^+^ in the T-cell panel, achieving an average accuracy of 0.96 on 596 cells. For the B-cell panel, we identified 13,858 ± 13,950 CD20^+^CXCR5^−^, 10,399 ± 8,510 CD20^+^CXCR5^+^, and 7,281 ± 4,336 CD79b^+^ cells with an average accuracy of 0.89 on 2,800 cells. To evaluate the impact of annotation budget on model performance, we retrained the classifier for the B-cell panel with different size of randomly sampled training set, and evaluated the performance on the same hold-out testing set. The model achieved an accuracy of 0.867, 0.879, 0.884, and 0.894 with 25%, 50%, 75%, and 100% of the annotations, indicating that the current amount of annotations was needed to obtain a reliable supervised classifier.

### Serial section alignment

T- and B-cell sections in the validation cohort were aligned to the corresponding H&E slide using manually chosen benchmarks. The affine transformation was performed using the “fitgeotrans” function in MATLAB R2019b (RRID:SCR_001622). The quality of registration was evaluated by (i) the Target Registration Error, which represents the distance between original and transformed points normalized by the diagonal length of an image (20); (ii) the concordance between densities of cancer cells and lymphocytes detected on H&E and IHC slides, respectively.

The averaged target registration error of aligned sections was 0.13% and the densities of cancer and lymphocytes detected on IHC significantly correlated to that on H&E sections (cancer: *R* = 0.69, *P* = 0.00079; lymphocyte: *R* = 0.76, *P* = 8.7 × 10^−5^; Supplementary Fig. S3A and S3B).

### Annotation of tertiary lymphoid structures and lymphoid aggregates

Lymphocyte clusters forming a structure were delineated by a senior pathologist on 10 digitized H&E slides from the LATTICe cohort. This manual identification was done while looking at both T-cell and B-cell serial IHC sections (Fig. 4A). Tertiary lymphoid structures (TLS) were marked as the aggregation of lymphocytes in which there was a clear zonality of T- and B-cell components, resulting in a structure. LAGs were marked as lymphocyte clusters without a distinguishable germinal center or admixed with stromal cells. Individual TLS and LAG were assigned to intratumoral, peritumoral, cancer hotspots and non-hotspot regions based on the location of the majority of its region. In total 52 TLSs were identified across 10 slides, with 49 in peritumoral IH, 2 in intratumoral IH, and 1 in non-hotspot regions. 1 out of 52 TLSs had the same area spanning intratumoral and peritumoral IH and was assigned the intratumoral IH. On the other hand, 98 LAGs were identified, with 66 in peritumoral IH, 17 in intratumoral IH, 9 in cancer hotspots and 6 in non-hotspot regions.

### Identification of individual immune hotspot

Disconnected IH was identified as cluster of 50×50 μm^2^ grids on the 10 H&E slides of the validation cohort. The spatial connectivity was assessed using the “clump” function in the R package “raster” with directions set as 8. Each individual IH was first segregated and indexed regardless of its distribution, then partially assigned to the peritumoral or intratumoral category based on labels of grids. Grids identified as part of TLS or LAG were excluded from the analysis. This resulted in 26 ± 11 individual intratumoral IH and 39 ± 12 individual peritumoral IH per slide.

### Cellular interaction analysis

We calculate the cellular interaction for each individual IH using the coordinates of immune cells within an individual IH as input. The spatially neighboring cells were connected using the Delaunay triangle graph, with the connections exceeding 250 μm excluded. The interactions between each pair of immune cell subsets were defined as the fractions of links between them among all the connections (21).

### Data availability

The transcriptomic data and the digital pathology images from the TCGA cohort can be accessed on the GDC portal (portal.gdc.cancer.gov, cohorts TCGA-LUSC and TCGA-LUAD). IHC data related to the validation cohort are available upon reasonable request.

### Code availability

Code and data used to generate figures and statistical information can be obtained at https://github.com/yuerua/t_b_spatial_IH. Scripts for implementing the SCCNN pipeline used for cell identification on the H&E and T-cell panel of the validation cohort are available at https://github.com/qalid7/compath. Scripts for the DRDIN pipeline used for cell identification on the B-cell panel of the validation cohort are available at https://github.com/pathdata/UNMaSk/tree/master/HE_cell_detection.

## Results

### High intratumoral immune hotspot score correlates with poor survival in LUSC

Image analysis and spatial statistics were used to identify spatially and histologically distinct immune-rich and cancer cell–rich areas in fresh frozen H&E slides (4) from 462 LUSC and 473 LUAD tumors from TCGA. We classified tissue neighborhoods into three compartments based on the densities of cancer or immune cells compared to the rest of the tissue: (i) Cancer hotspots: tissue regions enriched with cancer cells but not immune cells. (ii) Peritumoral IH: tissue regions enriched with immune cells but not cancer cells. (iii) Intratumoral IH, tissue regions enriched with both immune and cancer cells (Fig. 1A and B, see Materials and Methods; ref. 4).

Quantitative spatial scores *S*_*intra*/*immune*_, *S*_*intra*/*cancer*_, and *S*_*intra*/*tissue*_ were used to summarize the amount of intratumoral IH in relation to immune-rich regions, cancer-rich regions, and the entire tissue, respectively (Fig. 1B, see Materials and Methods; ref. 4). We observed a higher *S*_*intra*/*immune*_ in LUAD than LUSC (*P* = 0.011), and in females than males (*P* = 0.03). The *S*_*intra*/*cancer*_ was higher in females than males (*P* = 0.016). No statistically significant difference in the three spatial scores was found for groups categorized by other clinicopathologic characteristics, including age, smoking history, pack years, TMB, and stage (*P* > 0.05, Table 1).

**Table 1:** . Characteristics of the included patients and their association with the *S*_intra/immune_, *S*_intra/cancer_, and *S*_intra/tissue_.

Characteristics	Categories	Total *N* (%)	*S*_intra/immune_ *P* value	*S*_intra/cancer_ *P* value	*S*_intra/tissue_ *P* value
Histology (*N* = 935)	LUSC	462 (49.41%)	0.011	0.057	0.49
	LUAD	473 (50.59%)			
Age (*N* = 934)	≥65	566 (60.6%)	0.61	0.38	0.38
	<65	368 (39.4%)			
Sex (*N* = 935)	female	377 (40.32%)	0.03	0.016	0.21
	male	558 (59.68%)			
Smoking history (*N* = 903)	current reformed smoker for ⇐ 15 years	394 (43.63%)	0.36	0.17	0.32
	current reformed smoker for > 15 years	199 (22.04%)			
	current smoker	230 (25.47%)			
	lifelong nonsmoker	80 (8.86%)			
Pack years (*N* = 723)	≥35	478 (66.11%)	0.23	0.12	0.20
	<35	245 (33.89%)			
TMB (*N* = 902)	<3	347 (38.47%)	0.26	0.31	0.65
	≥3	555 (61.53%)			
Stage (*N* = 935)	ia	214 (22.89%)	0.075	0.31	0.20
	ib	257 (27.49%)			
	iia	114 (12.19%)			
	iib	159 (17.01%)			
	iiia	129 (13.80%)			
	iv	62 (6.63%)			

To determine the prognostic value of spatial scores, we carried out survival analysis in discovery and validation cohorts randomly split from the TCGA LUSC cohort, each containing 231 patients (Supplementary Table S2). Among the three scores, *S*_*intra*/*immune*_ was the only one significantly associated with the overall survival (OS) in both discovery (*P* = 0.019) and validation (*P* = 0.043) cohorts (Fig. 1C; Supplementary Fig. S4A and S4B), with high *S*_*intra*/*immune*_ indicative of an adverse outcome (threshold 0.326 determined in the discovery cohort). To examine the contingency of the prognostic value of *S*_*intra*/*immune*_ on the cohort split, we repeated the splitting of discovery and validation cohort for 100 times. In 36 iterations, *S*_*intra*/*immune*_ showed a significant prognostic effect (*P* < 0.05) in the validation cohort when dichotomized by the threshold optimized for the discovery cohort, with an averaged *P* value of 0.224 (SD, 0.261; 95% CI, 0.172–0.275). The percentage of significant iterations was higher than that of other well-known prognosticators including TNM stage (8%), age (4%), pack years (6%), and TMB (20%; Supplementary Table S3; refs. 22–25), signifying the discriminative ability of *S*_*intra*/*immune*_ to identify patients at high risk.

Multivariate analysis showed that *S*_*intra*/*immune*_ remained significant after accounting for age, stage, and smoking pack years in the validation cohort [*P* = 0.02; HR = 2.0 (1.12–3.7); Fig. 1D]. *S*_*intra*/*immune*_ was also significantly associated with poor outcome when tested separately in patients stratified on the basis of tobacco smoking history available in the TCGA clinical data, differentiating current reformed smokers for less than 15 years, current reformed smokers for more than 15 years, and current smokers (*P* < 0.05; Supplementary Fig. S5A). However, it was not significant in nonsmokers, potentially due to the small sample size (*P* = 0.18, *n* = 16; Supplementary Fig. S5A).

In LUAD tumors, *S*_*intra*/*immune*_ did not show significant prognostic value either in the validation cohort (*P* = 0.9, *n* = 236; Supplementary Fig. S6A) or within any of the smoking categories (*P* > 0.05; Supplementary Fig. S5B). The other two spatial scores *S*_*intra*/*cancer*_ and *S*_*intra*/*tissue*_were both not significant in the validation cohort (*P* = 0.094, *P* = 0.69, *n* = 236; Supplementary Fig. S6B and S6C).

This differential prognostic value of *S*_*intra*/*immune*_ between LUSC and LUAD pertaining to the spatial heterogeneity of lymphocyte distribution was not related to the overall immune infiltration in both NSCLC subtypes. Lymphocyte abundance, defined as the percentage of lymphocytes detected on the whole slide, was higher in LUAD than LUSC (*P* = 0.0018). A high level of lymphocyte abundance was associated with favorable OS in LUAD (*P* = 0.017) but not in LUSC (*P* = 0.08; Supplementary Fig. S7A and S7B). However, in multivariate analysis adjusted for stage, age and smoking pack years, lymphocyte abundance was not prognostic in either subtype (*P* > 0.05; Supplementary Fig. S7C and S7D). In addition, *S*_*intra*/*immune*_ did not correlate with lymphocyte abundance in LUSC (*R* = -0.081, *P* = 0.083; Fig. 1E). Tumors with high lymphocyte abundance and low *S*_*intra*/*immune*_ displayed accumulation of lymphocytes at the peritumoral region (Supplementary Fig. S8A), whereas in tumors with low lymphocyte abundance and high *S*_*intra*/*immune*_, the majority of tumor infiltrating lymphocytes were found within the tumor nest (Supplementary Fig. S8B). Patients with high lymphocyte abundance but low *S*_*intra*/*immune*_, indicating a preferential homing of lymphocytes to the peritumoral region, had significantly better prognosis than other patients [*P* = 0.00035, HR = 0.55 (0.4–0.76), Fig. 1F].

### Consistent upregulation of B-cell signatures associated with high intratumoral immune hotspot score

To investigate molecular heterogeneity underpinning intratumoral and peritumoral IH, we leveraged RNA-seq data to evaluate differentially expressed genes (DEG) in groups of patients with LUSC stratified by *S*_*intra*/*immune*_. Gene set enrichment analysis revealed that the Gene Ontology biological processes significantly enriched by these DEGs included immune response, inflammatory response, and activation of immune response (Fig. 2A). Immune related DEGs were exclusively B-cell markers, including *CD79B*, *MS4A1* (encoding *CD20*), and *CXCR5*, which were all upregulated in the high *S*_*intra*/*immune*_ group (Fig. 2B). *CXCR5* expression on B cells characterizes the subpopulation migrating into TLS, the organized lymphoid aggregates with morphologically distinct separation of T-cell and B-cell zones and serve as local sites of T-cell priming and B-cell maturation (26–30). The association between *S*_*intra*/*immune*_ and B-cell infiltration was cross-validated using seven established algorithms to estimate immune cell abundance, including Danaher (11), TIMER (10), CIBERSORT Absolute mode (12), MCP-counter (9), quanTIseq (13), EPIC (14), and xCell (15). Using these methods, we found a compelling pattern for an enrichment of B-cell populations in the high *S*_*intra*/*immune*_ group as compared with the low group according to the median (Fig. 2C).

In contrast with LUSC, *S*_*intra*/*immune*_ high group in LUAD showed a downregulation of B cell–related genes (*CD79b*, *FCER2*, *CXCR5*, *MS4A1*, *CD19*; Supplementary Fig. S2B). The enrichment analysis revealed that DEGs between high and low groups in LUAD were enriched for microtubule-based movement and cell-adhesion, unlike the predominance of immune-related pathways in LUSC (Supplementary Fig. S2A).

We next examined the cooccurrence of T- and B-cell genes and signatures in the high *S*_*intra*/*immune*_ LUSC group. B-cell memory genes such as *CD27* were significantly correlated with *FOXP3* expression (*R* = 0.8; Supplementary Fig. S9A), compared with a weaker correlation between *CD27* and *CD8A* (*R* = 0.55; Supplementary Fig. S9B), in line with a previous report (31). Both *CD27* and *FOXP3* were overexpressed in the high *S*_*intra*/*immune*_ group (Supplementary Fig. S9A and S9C). The MCP-counter B-lineage signature correlated with the T-cell signature but not the cytotoxic lymphocyte or CD8 T-cell signature, indicating that T-cell subsets more likely to coexist with infiltrating B cells were CD4^+^ T cells, and not effector T cells (Supplementary Fig. S9D).

We then investigated the association between *S*_*intra*/*immune*_ and potential indicators of immunotherapy response, including TMB, PD-1, PD-L1 expression, and the neoantigen load (1) in patients with LUSC. We observed a significantly lower TMB in *S*_*intra*/*immune*_ high group (*P* = 0.032, Supplementary Fig. S10A). On the other hand, there was no statistical difference in PD-1, PD-L1 expression, or number of clonal neoantigens between *S*_*intra*/*immune*_ high and low group (PD-1, *P* = 0.19; PD-L1, *P* = 0.12; neoantigen load, *P* = 0.29; Supplementary Fig. S10E, S10F and S10G). Consistent with previous results (24), low TMB was associated with adverse outcomes in LUSC (*P* = 0.014, Supplementary Fig. S10B). Despite the negative association between *S*_*intra*/*immune*_ and TMB, high *S*_*intra*/*immune*_ independently correlated with poor OS in the multivariate analysis including TMB, age, stage, and pack years [*P* = 0.022, HR = 1.6 (1.07–2.5); Supplementary Fig. S10C]. In addition, high TMB and low *S*_*intra*/*immune*_ codefined a group of patients with significantly prolonged survival (*P* = 0.0011; Supplementary Fig. S10D).

### Abundance of T- and B-cell subpopulations in intratumoral and peritumoral immune hotspots

To further investigate the distribution of B cells in relation to intratumoral and peritumoral IH as well as the interplay between T and B cells, we selected 10 LUSC tumors from the LATTICe cohort, with 6 displaying high *S*_*intra*/*immune*_ (see Materials and Methods). Guided by the gene expression analysis (Fig. 2B), we stained three consecutive sections, respectively, with a panel for B cells (CXCR5/CD79b/MS4A1, encoding CD20) and neoplastic cells (P40), a T-cell panel (CD4/CD8/FOXP3), and with H&E.

**Figure 3.**
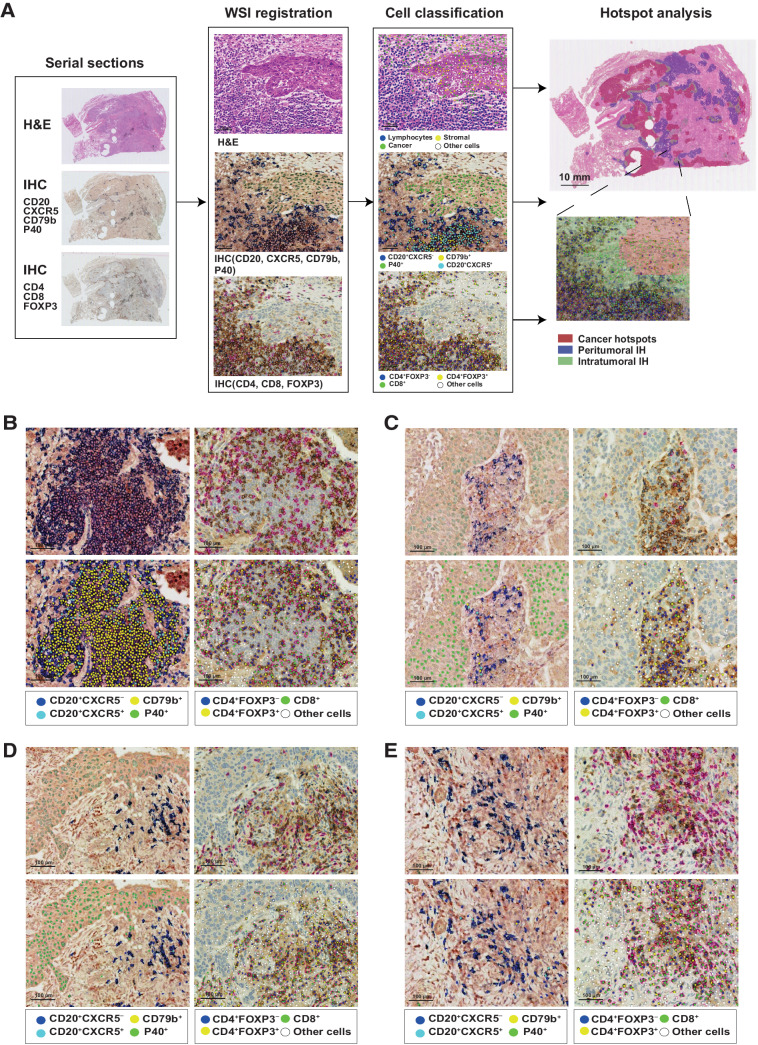
The deep learning pipeline to map T-cell and B-cell subsets at intratumoral and peritumoral IH. **A,** Framework of the deep learning pipeline for spatial analysis. The pipeline comprised 3 steps: serial section alignment, deep learning–based cell classification, and hotspots mapping. IHC staining with a B-cell panel (CD20/CXCR5/CD79b/P40) and a T-cell panel (CD4/FOXP3/CD8) was performed on serial sections, which were then aligned to the corresponding H&E section. Dominant B-cell subsets (CD20^+^CXCR5^−^, CD20^+^CXCR5^+^, CD79b^+^) and T-cell subsets (CD4^+^FOXP3^−^, CD4^+^FOXP3^+^, CD8^+^) were detected and classified by deep learning models and projected to the H&E slide. The magnified region example illustrates this entire framework, which combines immune cell detection from the B-/T-cell IHC sections together with hotspot identification on the corresponding H&E slide. **B–E,** Representative TLS, LAG, non-TLS/LAG intratumoral IH, and non-TLS/LAG peritumoral IH. Cell classification results are shown below the original image, with color codes for each cell type shown at the bottom. Scale bar, 100 μm.

The three sections were spatially aligned using the affine transformation based on manually selected landmarks (averaged misalignment distances: 43.33 μm; averaged target registration error: 0.13%, see Materials and Methods). Deep learning approaches were used to automate the identification of three dominant B-cell subsets (CD20^+^CXCR5^−^ cells, CD20^+^CXCR5^+^ cells, and CD79b^+^ cells; average accuracy: 0.89) and T-cell subsets (CD4^+^FOXP3^−^ T cells, CD8^+^ T cells, and CD4^+^FOXP3^+^ T regulatory (Treg) cells; average accuracy: 0.96, see Materials and Methods). The CD79b^+^ cells represented B cells with high CD79b expression and different expression levels of CD20 and CXCR5. CD20^+^CXCR5^−^ and CD20^+^CXCR5^+^ cells both showed low expression of CD79b. After the identification of B and T cells, we employed spatial statistics to quantify their distribution and colocalization patterns in intratumoral and peritumoral IH inferred from the corresponding H&E sections (Fig. 3A–E, see Materials and Methods).

**Figure 4.**
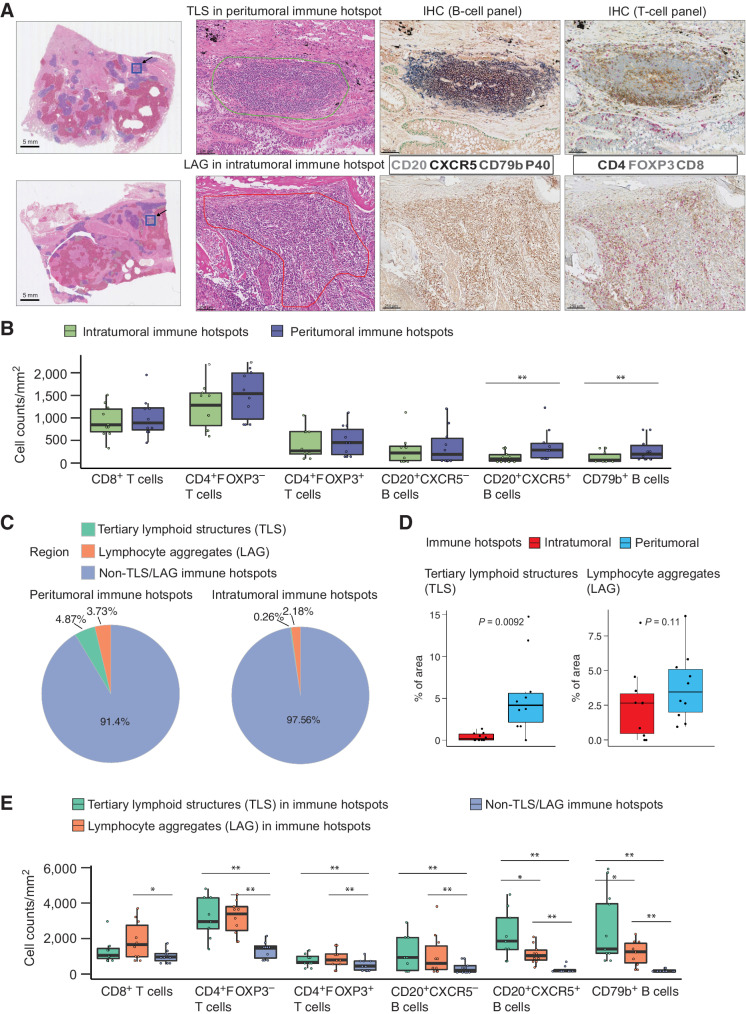
Lymphocyte composition and the presence of TLS at intratumoral and peritumoral IH. **A,** Example regions showing annotations of TLS at the IH and pathologically defined lymphoid aggregates without a prominent germinal center (LAG) at the intratumoral IH. **B,** Lymphocyte subset densities at intratumoral (*n* = 10) and peritumoral IH (*n* = 10). Cell densities were measured as the cell counts divided by the total area of IHs. **C,** Pie chart displaying the average percentages of peritumoral IH and intratumoral IH identified as TLS, LAG, and non-TLS/LAG IH. **D,** Densities of TLS and LAG at intratumoral (*n* = 10) and peritumoral IH (*n* = 10). **E,** Densities of B- and T-cell subsets in TLS (*n* = 10), LAG (*n* = 10), and non-TLS/LAG IH (*n* = 10). Statistical significance was evaluated by the Wilcoxon signed-rank test, followed by the Benjamini–Hochberg method for *P* value adjustment. *, *P* < 0.05; **, *P* < 0.01. The median is marked by the horizontal line; the first and third quartiles are shown as box edges; whiskers indicate the range of error.

In general, there was a higher density of T cells than B cells in the whole tissue, with CD4^+^FOXP3^−^ and CD8^+^ being the first and the second most abundant T-cell subsets (Supplementary Fig. S3C). There was no significant difference among densities of B-cell subsets (Supplementary Fig. S3C). The majority of T-cell subsets were found in the non-hotspot tissue regions, whereas the B-cell subsets displayed the highest percentages in the peritumoral IH (Supplementary Fig. S3D). We further compared immune cell compositions between intratumoral and peritumoral IH. Specific B-cell subsets, CD20^+^CXCR5^+^ cells and CD79b^+^ cells, displayed higher densities in peritumoral IH than in intratumoral IH (*P* < 0.05, Fig. 4B). There was no difference between densities of T-cell subsets in intratumoral and peritumoral IH (*P* > 0.05, Fig. 4B). The lower B-cell subset densities at the intratumoral IH compared with the peritumoral IH suggested that the concordant increase in B-cell signatures and proportions of intratumoral IH was due to recruitment of B cells to tumors with increased tumor–immune interface, rather than preferential homing of B cells to the intratumoral IH.

### TLSs account for a minor proportion of intratumoral and peritumoral immune hotspots

CXCR5 and CD79b have been recognized as markers associated with the TLS (28–30). To investigate the overlap of TLS with intratumoral and peritumoral IH, pathologists manually identified 52 TLSs with prominent germinal centers and 98 lymphoid aggregates (LAG) without a distinguishable germinal center or admixed with stromal cells from 10 H&E slides from the LATTICe cohort (Fig. 4A, see Materials and Methods).

The majority (49 of 52) TLSs were located in peritumoral IH, occupying a total of only 4.87% of the peritumoral IH area across all slides (mean per slide = 5.30%, SD = 4.62%), which was significantly higher than the proportion in intratumoral IH (2 TLS, total = 0.26%, mean per slide = 0.38%, SD = 0.49%, *P* = 0.0092; Fig. 4C and D). Conversely, LAGs were widespread across both compartments, with no significant difference in their proportions between intratumoral and peritumoral IH (intratumoral: *n* = 17, total = 2.18%, mean per slide = 2.66%, SD = 2.68%, peritumoral: *n* = 66, total = 3.73%, mean per slide = 3.93%, SD = 2.54%, *P* = 0.11; Fig. 4C and D). The rest of LAGs were found in cancer hotspots (*n* = 9) and non-hotspot regions (*n* = 6). The predominance of TLSs in peritumoral IH was consistent with the current paradigm that the majority of TLSs are located in the peritumoral area (32).

We observed a higher density of CD20^+^CXCR5^+^ and CD79b^+^ cells in TLS within IH than in non-TLS/LAG IH (*P* < 0.05; Fig. 4E). This is consistent with previous findings of the upregulation of *CXCR5* and *CD79b* in tumors with TLS present (28–30). TLS also contained a higher amount of CD4^+^ T cells, CD4^+^FOXP3^+^ Treg cells, and CD20^+^CXCR5^−^ B-cell subsets compared with non-TLS/LAG IH (*P* < 0.01, Fig. 4E), whereas only CD20^+^CXCR5^+^ and CD79b^+^ B cells were significantly different between TLS and LAG. Taken together, the majority of IHs are neither TLS nor LAG, based on their different B- and T-cell composition. We henceforth refer to these as non-TLS/LAG IH.

### B- and T-cell spatial interactions in non-TLS/LAG immune hotspots

**Figure 5.**
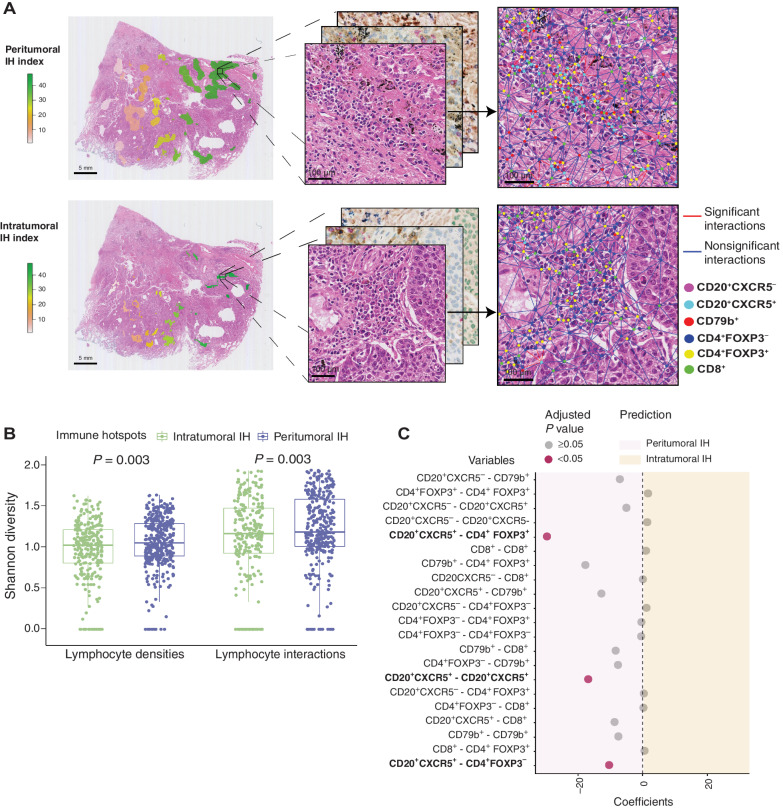
Diversity of lymphocyte interactions in intratumoral and peritumoral IH without TLS or LAG. **A,** Examples of lymphocyte interactions in individual peritumoral and intratumoral IH. Lymphocytes identified on the serial IHC slides were connected by the Delaunay triangle graph. Lymphocyte subsets are represented by dots of different colors. Interactions significant in the univariate logistic regression are denoted by red, otherwise blue. **B,** Shannon diversity of lymphocyte densities and interactions in individual peritumoral (*n* = 392) and intratumoral IH (*n* = 262). Statistical significance was evaluated by the Wilcoxon signed-rank test. **C,** Univariate logistic regression predicting peritumoral and intratumoral IH with individual lymphocyte interactions as variables, *n* = 654. The coefficients of the significant variables were all negative, suggesting a higher frequency of the interactions in peritumoral IH compared with intratumoral IH. *P* values were adjusted by the Benjamini–Hochberg method. Regions identified as TLS or LAG were excluded from the above analyses.

To further investigate the hazard underpinning intratumoral IH while excluding the confounding effect of TLS, we sought to resolve cell spatial interactions that differentiate non-TLS/LAG intratumoral and peritumoral IH. Using the Delaunay triangle graph (21), we developed a method for identifying individual non-TLS/LAG IH based on the connectivity of adjacent regions and construct cellular networks (Fig. 5A). Lymphocyte subset interactions were defined as the proportion of edges connecting two types of lymphocytes (see Materials and Methods). Both interactions and compositions of lymphocyte subsets were significantly more diverse in the peritumoral IH than in the intratumoral IH, as indicated by the higher Shannon index (*P* = 0.003; *P* = 0.003; Fig. 5B). Compared with intratumoral IH, peritumoral IH had higher frequencies of interactions between CD20^+^CXCR5^+^ and CD4^+^FOXP3^+^, CD4^+^FOXP3^−^, but not CD8^+^ cells (univariate logistic regression *P* < 0.05; Fig. 5C). On the other hand, none of the interactions involving CD79b^+^ B cells were observed to be significant despite the enrichment of CD79b^+^ B cells in peritumoral IH (*P* > 0.05, Fig. 5C), suggesting an independence between cell interactions and abundance.

### High abundance of B cells in non-TLS/LAG intratumoral immune hotspots was linked to a decrease in the CD8^+^/CD4^+^FOXP3^+^ ratio

**Figure 6.**
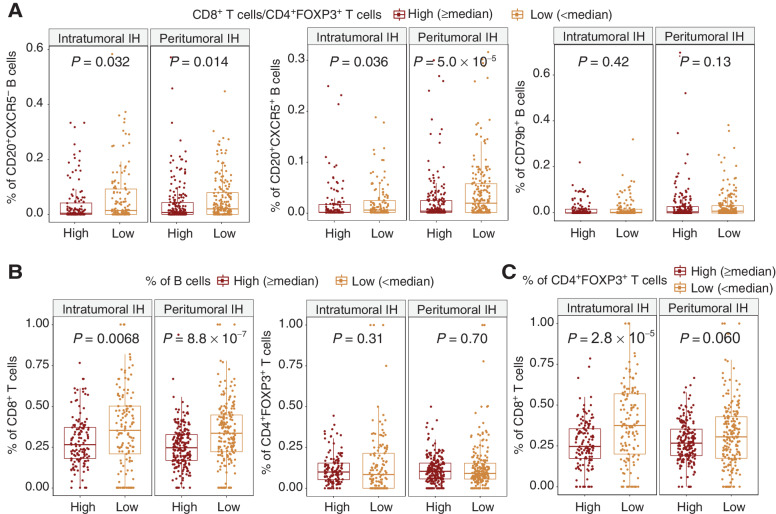
The relationship among B-cell, T-cell percentages and CD8^+^/CD4^+^FOXP3^+^ ratio in individual intratumoral IH and peritumoral IH without TLS or LAG. **A,** Percentages of B-cell subsets in individual intratumoral (*n* = 262) and peritumoral IH (*n* = 392) classified by CD8^+^/CD4^+^FOXP3^+^ ratio. The high and low groups were determined by the cut-off of median CD8^+^/CD4^+^FOXP3^+^ ratio in intratumoral and peritumoral IH, respectively. **B,** Percentages of CD8^+^ T cells and CD4^+^FOXP3^+^ Treg cells in individual intratumoral (*n* = 262) and peritumoral IH (*n* = 392) classified by the percentage of B cells. This B-cell percentage was calculated as the total count of all B-cell subsets divided by the total count of all 6 immune cell types. **C,** Percentages of CD8^+^ T cells in individual intratumoral (*n* = 262) and peritumoral IH (*n* = 392) classified by CD4^+^FOXP3^+^ Treg-cell percentages. Statistical significance was evaluated by the Wilcoxon rank sum test and adjusted by the Benjamini–Hochberg method.

We assessed the localized T-cell regulation by the CD8^+^/CD4^+^FOXP3^+^ ratio within individual non-TLS/LAG IH (see Materials and Methods). There was no difference between the CD8^+^/CD4^+^FOXP3^+^ ratio at intratumoral and peritumoral IH (intratumoral IH: mean = 4.66, SD = 7.09, peritumoral IH: mean = 3.72, SD = 4.3, *P* = 0.53). To further dissect the role of B cells on T-cell regulation within non-TLS/LAG IH, each non-TLS/LAG intratumoral and peritumoral IH was classified on the basis of its CD8^+^/CD4^+^FOXP3^+^ ratio. IH with low CD8^+^/CD4^+^FOXP3^+^ ratio had significantly higher percentages of CD20^+^CXCR5^−^ and CD20^+^CXCR5^+^ B cells (defined as the number of cells divided by the total count of 6 immune cell types in each IH) compared to IH with high CD8^+^/CD4^+^FOXP3^+^ ratio, regardless of their location (*P* < 0.05, Fig. 6A).

We then investigated whether the reduced CD8^+^/CD4^+^FOXP3^+^ ratio at B-cell–enriched IH was associated with the exclusion of CD8^+^ T cells or the recruitment of Treg cells. B-cell–enriched intratumoral and peritumoral IH had significantly lower percentages of CD8^+^ T cells compared with IH with low B-cell percentages (intratumoral IH: *P* = 0.0068, peritumoral IH: *P* = 8.8 × 10^−7^, Fig. 6B). In contrast, CD4^+^FOXP3^+^ T-cell percentage was not correlated with B-cell abundance in intratumoral IH or peritumoral IH (*P* > 0.5, Fig. 6B). In addition, we observed a strong negative correlation between CD8^+^ and CD4^+^FOXP3^+^ percentages in intratumoral (*P* = 2.8 × 10^−5^) but not in peritumoral IH (*P* = 0.060, Fig. 6C). Taken together, these findings suggest that while a generic pattern of decreased CD8^+^ cells but not Treg cells in B-cell–enriched IH is observed regardless of their location, Treg cells may have a bigger impact on the regulation of CD8^+^ cells in intratumoral IH compared with peritumoral IH, due to the presence of negative correlation between CD8^+^ T-cell and Treg abundance exclusively in intratumoral IH.

## Discussion

Heterogeneous spatial organization of tumor-infiltrating lymphocytes was associated with immune evasion and tumor evolution in NSCLC (2, 33). Specifically, the presence of TLS has been shown to correlate with prolonged survival in NSCLC and a variety of cancer types (28, 33–36). However, immune rich areas beyond TLS are not well studied. Immune-rich areas differ from TLS in terms of cellular densities, organizations, and spatial distributions (37–39). Understanding the role of these immune rich areas in cancer progression and treatment response may be crucial in improving therapies involving immune checkpoint blockade and induction of TLS against cancer (40). In this study, we integrated transcriptional profiling and computational pathology to deconvolve the spatial heterogeneity of immune rich areas identified by the hotspot analysis in NSCLC. We measured the proportion of IH colocalizing with tumor as a score *S*_*intra*/*immune*_. The score was a prognostic factor associated with unfavorable outcomes in LUSC independent of age, stage, and pack-years. Despite *S*_*intra*/*immune*_ being higher in LUAD than LUSC, the score did not show prognostic value or association with immune-related genes in LUAD. Such discrepancy adds to the current paradigm of the distinct biology and immune evasion mechanisms of the two histologic subtypes, such as the different prevalence of HLA LOH and clonal neoantigens (23). This finding highlights the importance of studying IHs beyond TLS and indicates that the prognostic value of lymphocyte clustering is nonexclusive to breast cancer.

The immune spatial score *S*_*intra*/*immune*_ may be a B-cell–enriched spatial signature, given the consistent upregulation of B-cell signatures in the TCGA cohort. It was later confirmed in the IHC experiments in the validation cohort based on B-cell accumulation at the IHs. In addition, LUSC with high *S*_*intra*/*immune*_ displayed a lower TMB, raising the possibility that increased IH located inside the tumor nest might characterize tumors undergoing clonal selection or adaptation to the immune microenvironment.

One of the potential reasons for *S*_*intra*/*immune*_ being an adverse survival indicator is that *S*_*intra*/*immune*_-high tumors had reduced peritumoral IH and therefore low TLS abundance, which has been associated with adverse outcome in NSCLC (33). TLS are characterized as organized lymphoid aggregates with a clear separation of T- and B-cell zones (28, 29). In the validation cohort, we found TLS frequently located outside the tumor nest, and contain higher densities of B cells and CD4^+^ T cells than intra and peritumoral IH. The majority of peritumoral and intratumoral IH were immune-rich areas without organized lymphoid structure. Given the minor proportion of TLS within both intratumoral and peritumoral IH, it is unlikely that *S*_*intra*/*immune*_ is a mere reverse approximate of TLS abundance. Comparisons between non-TLS/LAG intratumoral and peritumoral IH showed increased interactions between CD20^+^CXCR5^+^ B and CD4^+^ T cells, Treg cells in the non-TLS/LAG peritumoral IH. B cells have been shown to present antigen and to activate CD4^+^FOXP3^−^ T cells at the interfollicular region in lymph nodes and within TLS (41, 42). It is possible that CD20^+^CXCR5^+^ B cells in non-TLS/LAG peritumoral IH also exhibit such proinflammatory functions, contributing to the antitumor immune response in peritumoral IH. The enhanced interactions between CD20^+^CXCR5^+^ B cells and Treg cells suggested that B cells in peritumoral IH were subjected to stronger regulation, potentially due to the accumulation of tumor-specific autoantibodies in peritumoral IH (43).

Furthermore, both non-TLS/LAG peritumoral and intratumoral IH exhibit a decreased CD8^+^ T-cell percentage and CD8^+^/CD4^+^FOXP3^+^ ratio in response to the increased proportions of CD20^+^CXCR5^−^ and CD20^+^CXCR5^+^ B cells. In addition, an increase in Treg percentage was associated with decreased CD8^+^ T-cell proportions in non-TLS/LAG intratumoral IH but not in peritumoral IH (Fig. 6C). This negative relationship in intratumoral IH may indicate an immune escape strategy of tumor cells, where some tumor islands favor the recruitment of Treg over cytotoxic T cells, leading to a low-CD8^+^ high-Treg intratumoral IH phenotype (44, 45). Taken together, these results suggest an immunosuppressive TME imposed by the accumulation of B cells and T-cell regulation, with cytotoxic T cells subjected to greater regulation in intratumoral IH than in peritumoral IH. Therapeutic agents targeting B cells and Treg cells, such as rituximab and daclizumab, might skew the balance between intratumoral and peritumoral IH toward an immunogenic microenvironment.

**Figure 7.**
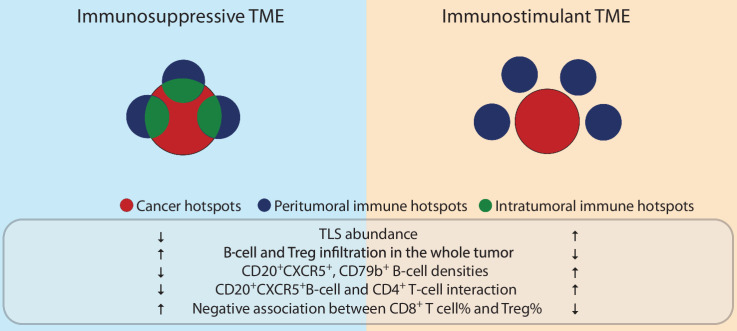
Immune spatial balance in LUSC. Spatial positioning of immune rich areas can reflect an immunosuppressive or an immunostimulant microenvironment. Increased intratumoral IH compared with peritumoral IH may reflect an immunosuppressive TME, characterized by decreased TLS abundance, lower densities of CD20^+^CXCR5^+^, and CD79b^+^ B-cell densities, decreased interactions between CD20^+^CXCR5^+^ B cells and CD4^+^ T cells, and the presence of a negative correlation between CD8^+^ T-cell and Treg cell abundance. Also, high ratio of intratumoral IH is coupled with increased B-cell and Treg infiltration in the whole tumor. Cancer hotspots, peritumoral IHs, and intratumoral IHs are denoted by red, blue, and green colors, respectively. An IH can consist of peritumoral and intratumoral compartments, denoted as circles with a combination of green and blue.

This study has several limitations. The IHC panel was designed based on the DEGs inferred from the TCGA cohort, which did not include markers for the functionality of T cell subsets such as PD-1, ICOS, and GZMB (37). It is possible that CD8 T cells residing in intratumoral IH are prone to exhaustion and are expected targets of immune checkpoint blockade therapy. Likewise, this panel was unable to reveal the potential immunosuppressive role of B cells. For example, *in vitro* experiments demonstrated that B regulatory cells promoted the expression of FOXP3 and CTLA4 in Treg via secretion of TGFβ and cell–cell contact (46). In addition, naïve B cells and follicular lymphoma B cells have been shown to induce the conversion of CD4^+^ T cells to Treg in a cell–cell contact manner (47, 48), which potentially contributed to the increased signal of Treg observed in the TCGA transcriptomic data. Also, B cells in intratumoral IH can possibly acquire an IL10-secreting phenotype that displays an immunoregulatory role (49). To fully appreciate the components, functionalities, and spatial interactions of lymphocytes and myeloid cells in intra and peritumoral IH, future studies are warranted. Also, the heterogeneous distribution of IH with respect to tumor glands might be attributed to a diverse immune evasion capability of tumor clones, as indicated by the branching evolution and tissue architecture of invasive glandular tumors (50). Another caveat is the identification of TLS, which was dependent on the variable choices of markers and morphologic criteria across studies (33). Also, the shape and structure of TLS may be varied by the sectioning angle. As a result, despite best efforts, the annotated TLS and LAG regions in the validation cohort may exclude partial TLS regions and immature TLS. However, the presence of CD20^+^CXCR5^+^ and CD79b^+^ was consistently observed in non-TLS/LAG IH, indicating that the two types of B cells preferentially reside in the peritumoral IH with respect to intratumoral IH regardless of the presence of TLS. In addition, further investigation on the distribution of IHs in preinvasive lesions has the potential to benefit the patient risk assessment, as immune surveillance in carcinoma *in situ* has been associated with lesion regression (51).

In conclusion, we propose a new spatial signature, *S*_*intra*/*immune*_, of protumor response orchestrated by B cells and T cells, as the balance between intratumoral and peritumoral IH (Fig. 7). Our study demonstrated a negative association between *S*_*intra*/*immune*_ and OS in LUSC. Increased *S*_*intra*/*immune*_ was correlated with an upregulated B-cell signature. Although limited by sample size, our preliminary results indicated the accumulation of CD20^+^CXCR5^−^/CD20^+^CXCR5^+^/CD79b^+^ B cells and the minor proportion of TLSs in IH. The interplay between CD8^+^ T cells, Tregs, and B cells underlying the spatial clustering pattern of lymphocytes characterized a protumor microenvironment in the intratumoral IH that may fuel the progression of LUSC.

## Supplementary Material

Supplementary Data Table S3 and Fig. S1-S10

Table S1 Differentially expressed genes in TCGA LUSC and LUAD patients with high versus low S_intra/immune

Table S2 Lists of TCGA LUSC and LUAD cases in the discovery and validation cohort
