# Supplementary material for: Spatial Positioning of Immune Hotspots Reflects the Interplay between B and T Cells in Lung Squamous Cell Carcinoma
Source: Cancer Res. 2023 Feb 28;83(9):1410–25. doi: 10.1158/0008-5472.CAN-22-2589 (PMC10152235; doi:10.1158/0008-5472.CAN-22-2589)
Supplement: Supplementary Data — Table S3 and Fig. S1-S10 [file can-22-2589_supplementary_data_suppst3_sf1-sf10.docx]

**Spatial positioning of immune hotspots reflects a balance of B and T cell interplays in Lung Squamous Cell Carcinoma**

**Supplementary data**

Table S3, Figures S1-S10

**Table. S3 | Prognostic assessment of clinical parameters across 100 random splits of discovery and validation cohorts in TCGA LUSC patients.** Cases without parameter available were excluded. The cohort was split by a 1:1 ratio. The numerical parameters ($S_{intra/immune}$, age, pack years, lymphocyte%, and TMB) were dichotomized by the threshold optimized for the discovery cohort, followed by a log-rank test performed in the validation cohort to determine the statistical difference in the overall survival of patients stratified by the dichotomized parameters. The TNM stage was assessed as a categorical parameter in the validation cohort.

|  | Threshold determined in the discovery cohort | p value of the survival test in the validation cohort | % of iterations with p<0.05 | 95% CI of p value |
| --- | --- | --- | --- | --- |
| $S_{intra/immune}$ | 0.264 +- 0.103 | 0.224 +- 0.261 | 36% | 0.172-0.275 |
| TNM Stage | - | 0.411 +- 0.273 | 8% | 0.356-0.465 |
| Age | 69.38 +- 5.77 | 0.491 +- 0.279 | 4% | 0.436-0.546 |
| Pack years | 57.86 +- 22.10 | 0.515 +- 0.295 | 6% | 0.456-0.573 |
| TMB | 3.02 +- 1.84 | 0.338 +- 0.307 | 20% | 0.277-0.399 |

**Figure S1 | Validation of H&E image analysis for NSCLC.** Correlation between H&E-based estimate of cancer cell (**A**), stromal cell (**B**), and immune cell (**C**) percentages by automated image analysis, pathological scores, copy-number based tumor purity measures ABSOLUTE, and gene expression-based signatures ESTIMATE, TIMER and MCP-counter. Different numbers of samples were used in each comparison due to data availability.

**Figure S2 | DEGs related to** $S_{intra/immune}$ **in TCGA LUAD. A.** Gene Ontology biological processes found to be enriched in the DEGs according to patient groups stratified by $S_{intra/immune}$. The red line denotes the ratio of the number of DEGs in a pathway to the total number of genes in that pathway. **B.** Volcano plot showing DEGs in the high-$S_{intra/immune}$ group compared with the low group. Genes with logFC smaller than 1 were excluded. Genes involved in immune responses are highlighted in yellow.


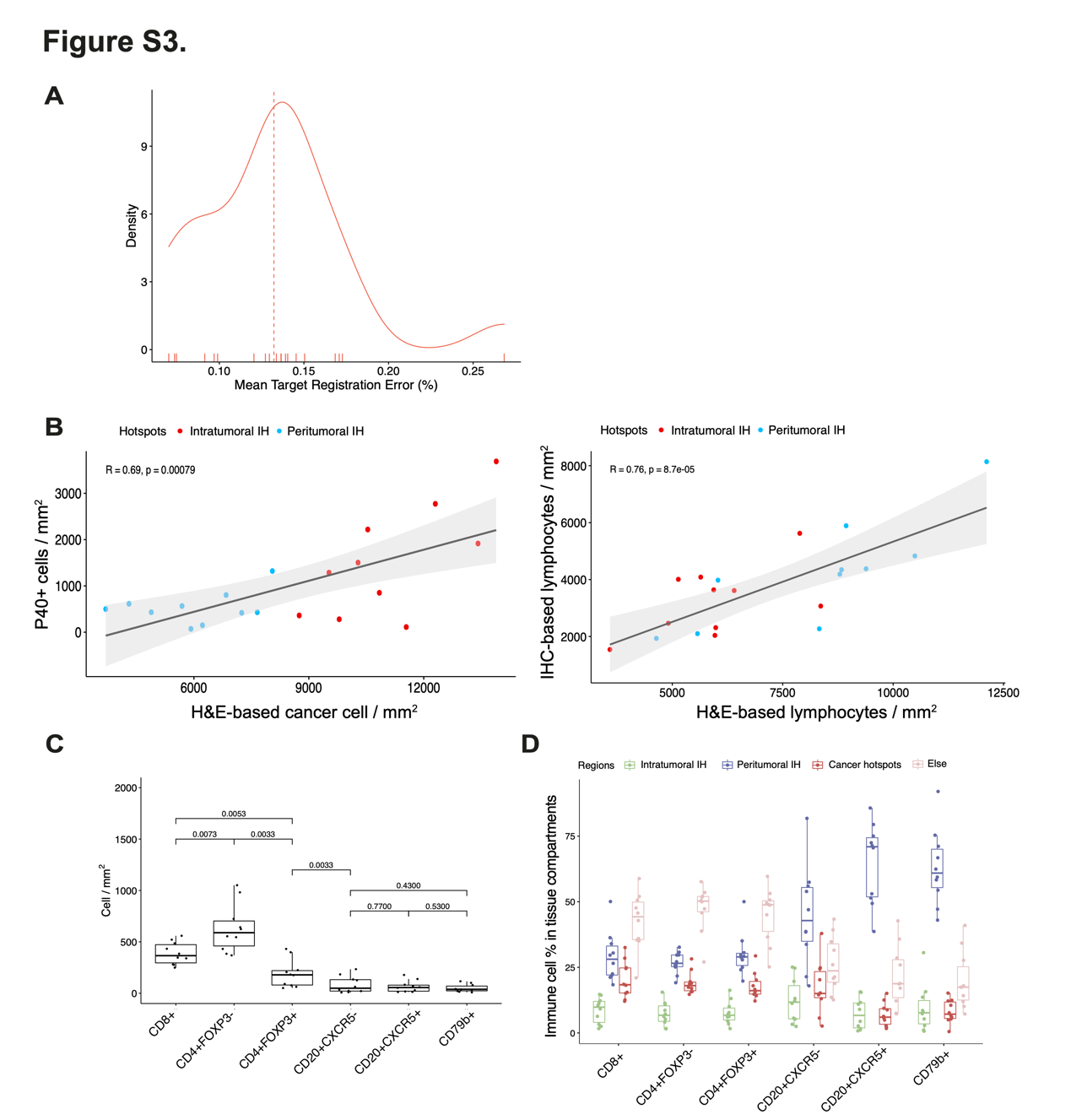


**Figure S3 | Assessment of registration quality and the lymphocyte densities of serial H&E and IHC sections of 10 LUSC patients. A.** The distribution of averaged Target Registration Error for registered slides. TRE is computed as the Euclidean distance between manually picked landmarks on matching slides normalized by the image diagonal. A total of 216 landmarks were chosen for 20 pairs of slides. The average distance between landmarks was 43.55 µm, giving an average TRE of 0.13%. **B.** Correlation between the density of lymphocytes and cancer cells on H&E and IHC sections. Only cells at intratumoral and peritumoral immune hotspots were included. **C.** Densities of lymphocyte subsets in the whole slide. Statistical significance was determined using the Wilcoxon signed-rank test. **D.** Percentages of immune cell subsets in different tissue compartments. P values adjusted by the Benjamini-Hochberg method were shown above each comparison.

**Figure S4 | Prognostic significance of** $S_{intra/cancer}$ **and** $S_{intra/tissue}$ **in TCGA LUSC patients. A, B**. Kaplan-Meier curves illustrating the prognostic significance of $S_{intra/cancer}$ and $S_{intra/tissue}$ in TCGA LUSC validation cohort (n=231). The cutoff was optimized for the discovery cohort and applied to the validation cohort.


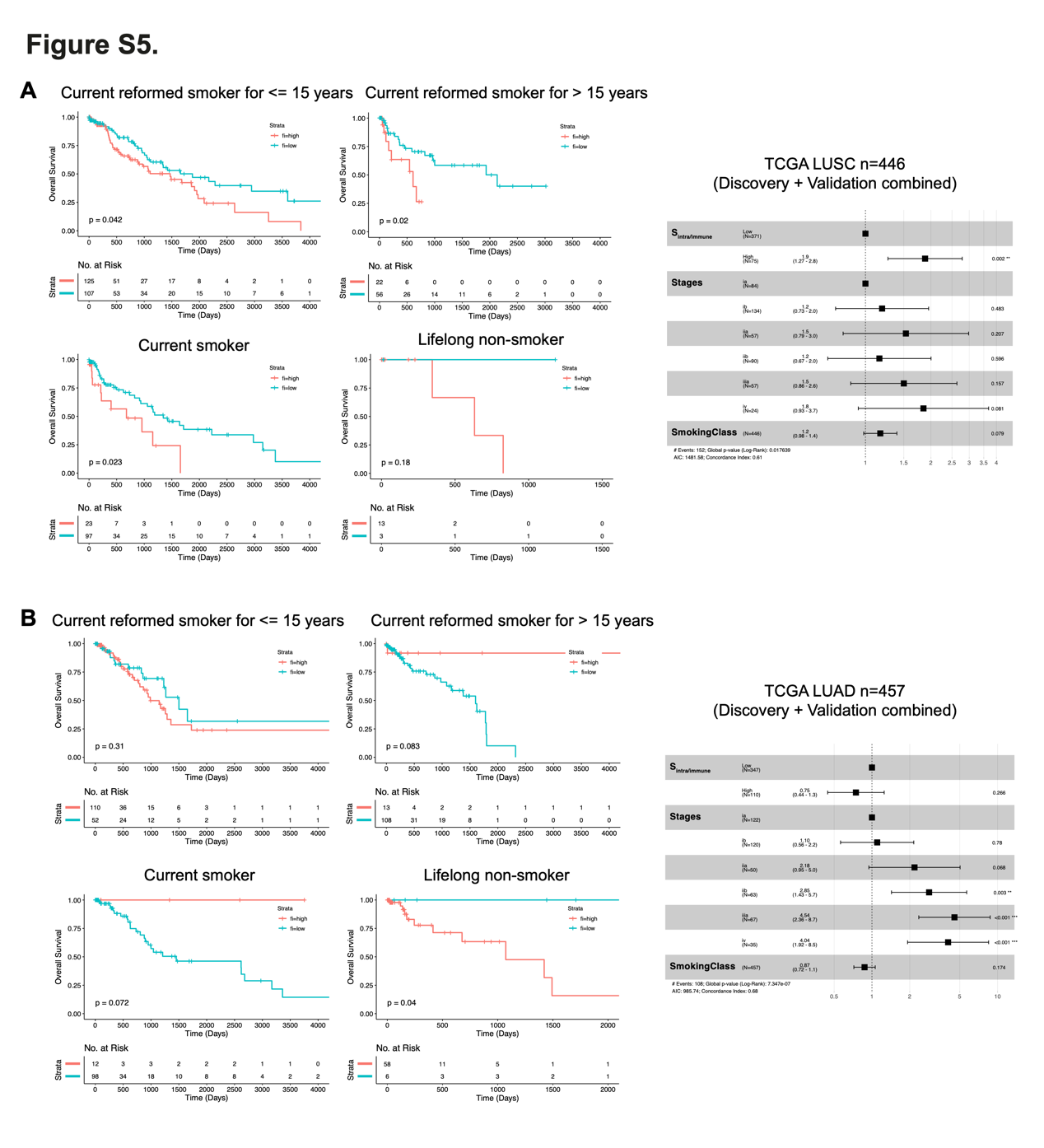


**Figure S5 | Prognostic significance of** $S_{intra/immune}$ **in TCGA NSCLC patients with different smoking histories. A.** Kaplan-Meier curves illustrating the difference in OS stratified by $S_{intra/peri}$ in TCGA LUSC patients of 4 different smoking categories (n = 446), and forest plot to show the prognostic value of $S_{intra/immune}$ in multivariate models adjusted for stage and smoking categories in the discovery and validation cohorts. Patients without defined smoking categories were excluded. **B.** Results of the same survival analysis repeated for TCGA LUAD patients (n = 457).

**Figure S6 | Prognostic significance of three immune scores** **in TCGA LUAD patients. A.** Kaplan-Meier curves showing the differences in OS stratified by $S_{intra/immune}$ in the validation cohort of TCGA LUAD (n = 236). The cutoff was optimized for the discovery cohort and applied to the validation and the entire cohort. **B, C.** Kaplan-Meier curves for $S_{intra/cancer}$ and $S_{intra/tissue}$.


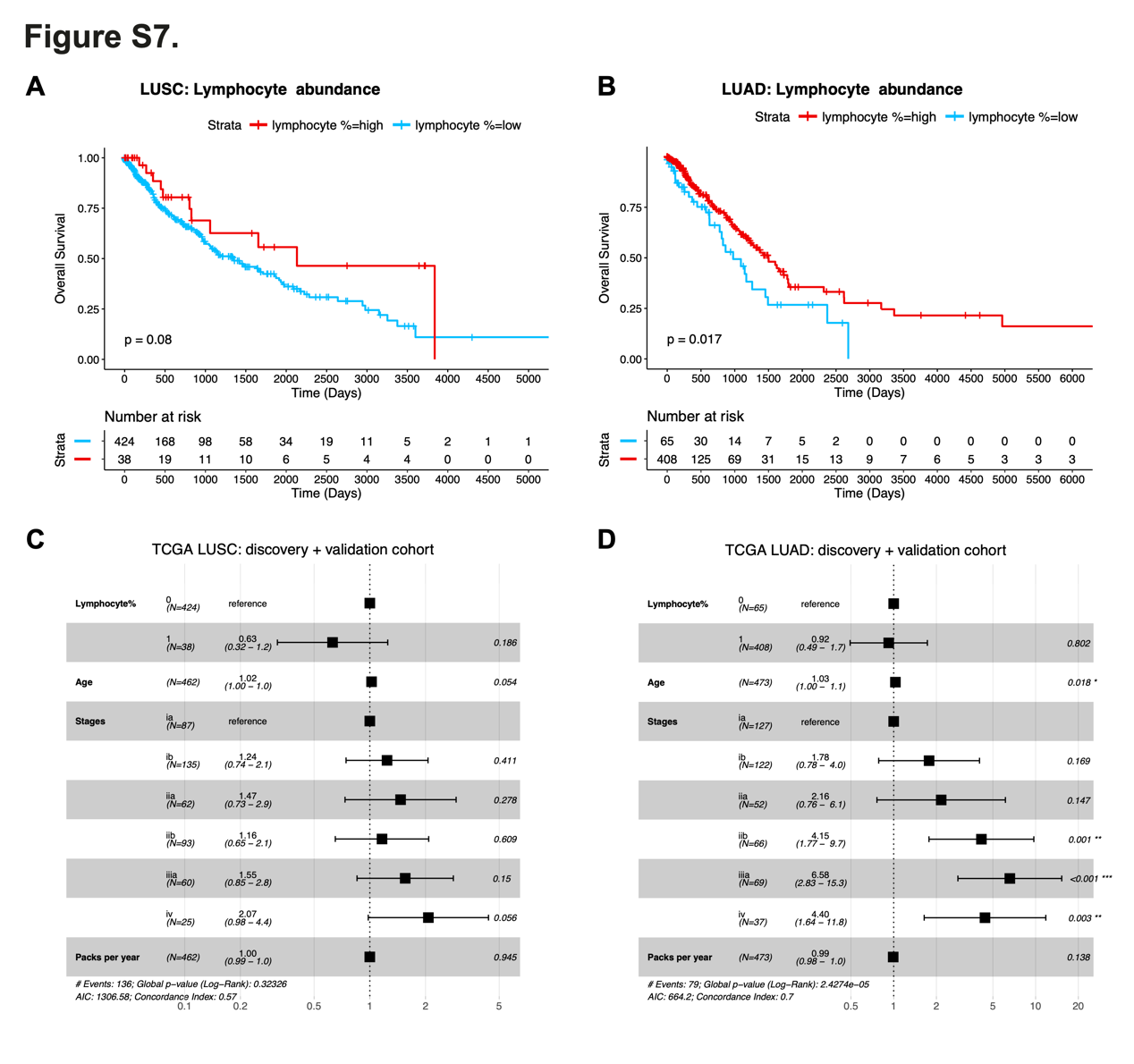


**Figure S7 | Survival test of lymphocyte abundance in TCGA LUSC and LUAD cohort.** **A, B**. Kaplan-Meier curves to illustrate the difference in OS in TCGA LUSC (n = 462) and LUAD (n = 473) patients stratified by lymphocyte abundance. **C, D.** Forest plots to show the prognostic value of lymphocyte abundance in multivariate models adjusted for stage, age, and smoking pack years in TCGA LUSC and LUAD patients.

**Figure S8 | Example slides with discordant lymphocyte% and** $S_{intra/immune}$ **from the TCGA LUSC cohort. A.** LUSC slides with high lymphocyte% and low $S_{intra/immune}$, displaying a high number of peritumoral IH (blue) at the peritumoral region. **B.** LUSC slides with low lymphocyte% and high $S_{intra/immune}$. The majority of IH were intratumoral IH (green). Zoomed-in regions showed areas enriched with peritumoral IH (**A**) and intratumoral IH (**B**).


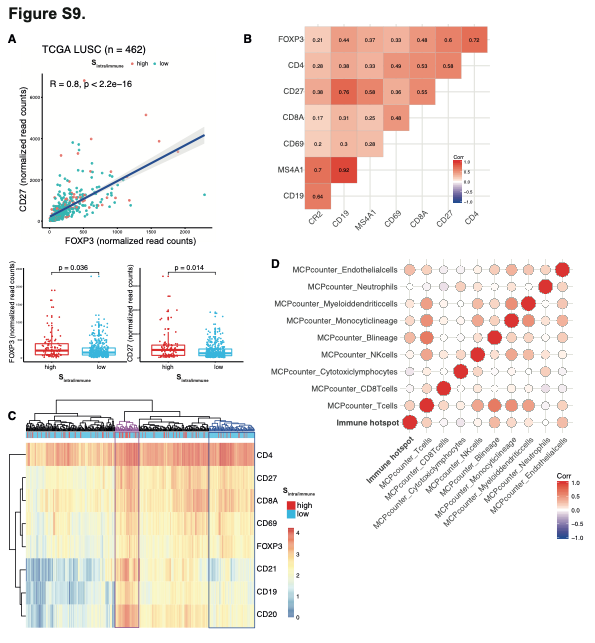


**Figure S9 | Immunological phenotype of immune hotspots orchestrated by infiltrating B cells and T regulatory cells. A**. Scatter plot to show the correlation between *CD27* and *FOXP3* gene expression (measured by read counts after quantile normalization) in 462 TCGA LUSC. r referred to Pearson’s correlation coefficient; shading shows the 95% confidence interval. **B.** Correlation heatmap to illustrate the relationship and co-clustering of B cell and T cell related genes. **C**. Gene expression heatmap for the same genes as in **B.** **D.** Correlations among immune hotspot and MCP-counter gene signatures.


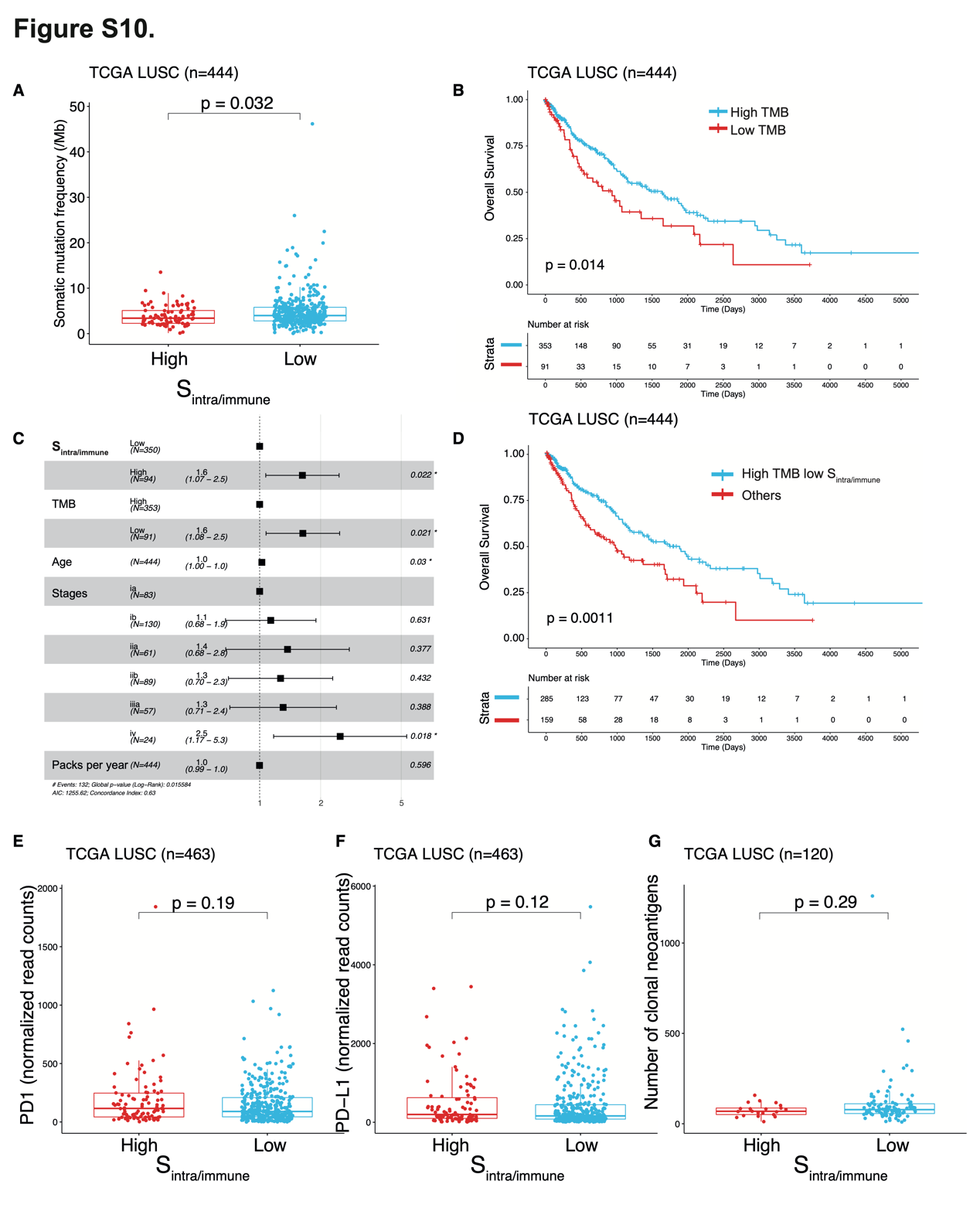


**Figure S10 | The association between** $\boldsymbol{S}_{\boldsymbol{intra/immune}}$ **and** **TMB, PD1, PD-L1, and neoantigens in LUSC.** **A.** Increased TMB in $\boldsymbol{S}_{\boldsymbol{intra/immune}}$ low patients. **B.** Kaplan-Meier curves to illustrate the difference in OS in LUSC patients stratified by TMB. **C.** Forest plot to show the prognostic value of $\boldsymbol{S}_{\boldsymbol{intra/immune}}$ in multivariate models adjusted for stage, age, and smoking pack years in the entire cohort. **D.** Kaplan-Meier curves to illustrate the difference in OS in LUSC patients stratified by both $\boldsymbol{S}_{\boldsymbol{intra/immune}}$ and lymphocyte percentage, with the stratification of the entire cohort shown. **E-G.**  Comparisons of PD-1, PD-L1 and neoantigen load in $\boldsymbol{S}_{\boldsymbol{intra/immune}}$ high and low group.
